# Two Factor Reprogramming of Human Neural Stem Cells into Pluripotency

**DOI:** 10.1371/journal.pone.0007044

**Published:** 2009-09-18

**Authors:** Mark E. Hester, SungWon Song, Carlos J. Miranda, Amy Eagle, Phillip H. Schwartz, Brian K. Kaspar

**Affiliations:** 1 The Research Institute at Nationwide Children's Research Institute, Columbus, Ohio, United States of America; 2 Molecular, Cellular, Developmental Biology, The Ohio State University, Columbus, Ohio, United States of America; 3 National Human Neural Stem Cell Resource, Children's Hospital of Orange County Research Institute, Orange, California, United States of America; University of Washington, United States of America

## Abstract

**Background:**

Reprogramming human somatic cells to pluripotency represents a valuable resource for the development of *in vitro* based models for human disease and holds tremendous potential for deriving patient-specific pluripotent stem cells. Recently, mouse neural stem cells (NSCs) have been shown capable of reprogramming into a pluripotent state by forced expression of Oct3/4 and Klf4; however it has been unknown whether this same strategy could apply to human NSCs, which would result in more relevant pluripotent stem cells for modeling human disease.

**Methodology and Principal Findings:**

Here, we show that OCT3/4 and KLF4 are indeed sufficient to induce pluripotency from human NSCs within a two week time frame and are molecularly indistinguishable from human ES cells. Furthermore, human NSC-derived pluripotent stem cells can differentiate into all three germ lineages both *in vitro* and *in vivo*.

**Conclusions/Significance:**

We propose that human NSCs represent an attractive source of cells for producing human iPS cells since they only require two factors, obviating the need for c-MYC, for induction into pluripotency. Thus, *in vitro* human disease models could be generated from iPS cells derived from human NSCs.

## Introduction

Numerous laboratories have demonstrated that human and mouse somatic cells can be reprogrammed into pluripotent stem cells or iPS cells by the forced expression of a characterized set of transcription factors (Oct4, Sox2, c-Myc, Klf4, Nanog, and Lin28) in various combinations [1]–[10]. Oct4, Sox2, c-Myc, and Klf4 were the four original factors that were capable of converting mouse and human fibroblasts into iPs cells [1], [3], [5], [11], [12]. More recently, murine liver, stomach [8], lymphocyte, β[13], and murine neural stem cells (NSCs) [14]–[16] were also capable of iPs induction. Since murine NSCs already express high levels of Sox2 [14]–[16], it was tested whether these cells could be reprogrammed into iPS cells by only a few critical factors. Indeed, it was shown that Oct4 and Klf4 could reprogram murine NSCs at an efficiency of 0.11%, similar to the reprogramming rate of murine fibroblasts with the original four factors with antibiotic selection [14]. More recently, the forced expression of Oct4 alone was shown sufficient to reprogram murine NSCs, albeit at a low efficiency of 0.014% [15]. Since murine NSCs have been “primed” with several of the factors originally discovered to reprogram fibroblasts into iPS cells, they represent an attractive source of starting material for iPS cell induction studies.

Here we tested whether human NSCs could be reprogrammed into iPS cells utilizing a similar strategy as described above since they represent a more clinically relevant source of cells for basic studies and modeling human disease. Human NSCs can be isolated and cultured from fetal, adult, as well as post-mortem brain tissues, and can differentiate into astrocytes, oligodendrocytes, and neurons [17], [18]. Similar to murine NSCs, human NSCs also express high levels of SOX2 and may therefore only require a limited set of factors for induction into pluripotency. Here we show that human NSCs indeed can be reprogrammed into iPS cells by ectopic expression of OCT3/4 and KLF4. Furthermore, we have demonstrated by several rigorous methods that human NSC-derived iPS cells are molecularly identical to hESCs.

## Materials and Methods

### Cell culture and differentiation

Fetal human NSCs, isolated from the frontal brain cortex of a 28 week term fetus, (SCP-27, P1) were obtained from the National Human Neural Stem Cell Resource (NHNSCR, Orange, CA). Proliferating cells were cultured in DMEM/F12 supplemented with 1% N2 (Invitrogen, Carlsbad CA), 10% BIT-9500 (Stem Cell), 1% penicillin, streptomycin, amphocterin cocktail, EGF (20 ng/ml, Peprotech), and FGF-2 (20 ng/ml, Peprotech). Cells were grown on polyornithine and laminin coated dishes and passaged 1:2 with PBS++ (PBS with 1% BSA). All experiments performed with human NSCs were from passage 10–12. To differentiate NSCs into neurons, proliferating media was replaced with a similar media as described above without growth factors and supplemented with all-trans Retinoic acid (Sigma) at 2 uM and forskolin (Sigma) at 5 uM. To induce astrocytic differentiation, NSCs were cultured in DMEM/F12 supplemented with 1% N2 and 10% fetal bovine serum. Oligodendrocyte differentiation was induced by culturing the cells in DMEM/F12, 1% N2, and IGF-1 (200 ng/ml). In all conditions, cells were allowed to differentiate for 1 week.

The UC06 (HSF6) human ES cell line (P62) was obtained from the National Stem Cell Bank (NSCB), and the adipose derived mesenchymal stem cell (AD-MSC) line was generously obtained from Dr. Jeffrey Gimble from the Penington Biomedical Research Institute.

To induce mesoderm and endoderm lineages from iPS cells, cells were grown as embryoid bodies (EB) in DMEM/F12 with 10% fetal bovine serum for 2 weeks. EBs were then plated to 12 well culture dishes to allow them to adhere to form a monolayer. To induce ectoderm formation, EBs were cultured in DMEM/F12 and 1% N2 supplemented with retinoic acid (2 uM) and forskolin (5 uM).

### Viral production and human iPS induction from human NSCs

Retroviral vectors containing cDNAs for OCT3/4 and KLF4 were obtained from Addgene (Cambridge, MA) and transfected, along with packaging plasmids CMV Gal-Pol and VSVG, into human embryonic kidney carcinoma 293 cells (HEK-293) using the CaCl_2_ transient transfection method. Supernatants were collected for 3 consecutive days beginning 48 hours after transfection. Virus was concentrated by centrifugation at 90,000x g for 2 hours at 4°C, and resuspended in 200 µl PBS. Viral titers were determined to be approximately 10^8^ viral particles/ml based on immunocytochemical analysis. 10^6^ human NSCs (passage 10) were infected with retroviruses encoding OCT3/4 alone or OCT3/4 and KLF4 at an MOI of 10 in human NSC media. In two-factor reprogramming experiments, 80% of cells were infected with both OCT3/4 and KLF4. Similar targeting efficiencies were obtained when OCT3/4 was transduced alone.

### Bisulfite sequencing

DNA was isolated from human NSCs, ESCs and iPS cells and treated with bisulfite using the EpiTect Bisulfite Kit (Qiagen) according to manufacturer's instructions. Amplified products were purified using gel filtration columns (Qiagen), cloned into the pCR2.1-TOPO vector (Invitrogen), and sequenced with M13 forward and reverse primers.

### Alkaline phosphatase staining

Alkaline phosphatase staining was performed with the Alkaline Phosphatase Detection Kit (Millipore) according to manufacturer's instructions.

### qPCR and RT-PCR analysis

RNA was harvested from proliferating HSF6 hESCs (P67) and MSCs (P10). Total RNA was reverse transcribed with RT^2^ First Strand Kit (SABiosciences) according to the manufacturer's instructions. Primer sequences are listed in Table S1. Real-time quantitative PCR reactions were performed using RT^2^ Real-Time SYBR Green/Rox PCR Master Mix (SABiosciences) and run on an RT^2^ Profiler PCR Array for Human Embryonic Stem Cells (SABiosciences).

### Immunofluorescent analysis

Cells were fixed with 4% PFA, followed by three rinses of TBS for 5 minutes, and then blocked with 10% donkey serum with 0.1% Triton X-100. All primary antibodies were diluted in blocking solution and incubated overnight at 4°C. Primary antibodies utilized are listed in Table S1. Nuclei were counterstained with DAPI. All images were captured using a Zeiss LSM510-META confocal laser-scanning microscope (Zeiss, Thornwood, NY).

### Teratoma analysis

Teratomas were generated from iPS clones 1 and 3 by subcutaneous injection of 1×10^6^ cells into the dorsal flank of isoflurane-anesthetized athymic nude mice (Jackson Labs). Teratomas were recovered 3–4 weeks post-injection, fixed for 24 hours in 10% formalin, embedded in paraffin, and sectioned at a thickness of 10 µM. Sections were stained for hematoxylin and eosin.

### Karyotype analysis

Karyotyping of human NSC-derived iPS lines was performed at the Molecular Cytogenetics shared resource at the Ohio State University Comprehensive Cancer Center. Karyotyping was performed on iPS lines 1 and 2.

## Results

### Human NSCs Express Genes Associated With Pluripotency

ESCs are maintained in a state of pluripotency by a precise regulation of numerous intrinsic and extrinsic factors. In hESCs, Fibroblast growth factor (FGF), activin, and Bone morphogenetic signaling (BMP) signaling have been shown to be critical in maintaining self-renewal [19]. In addition, intrinsic factors such as OCT3/4, SOX2, and NANOG are generally regarded as the central core of genes that keep hESCs in an undifferentiated state capable of indefinite self-renewal [20].

**Figure 1:**
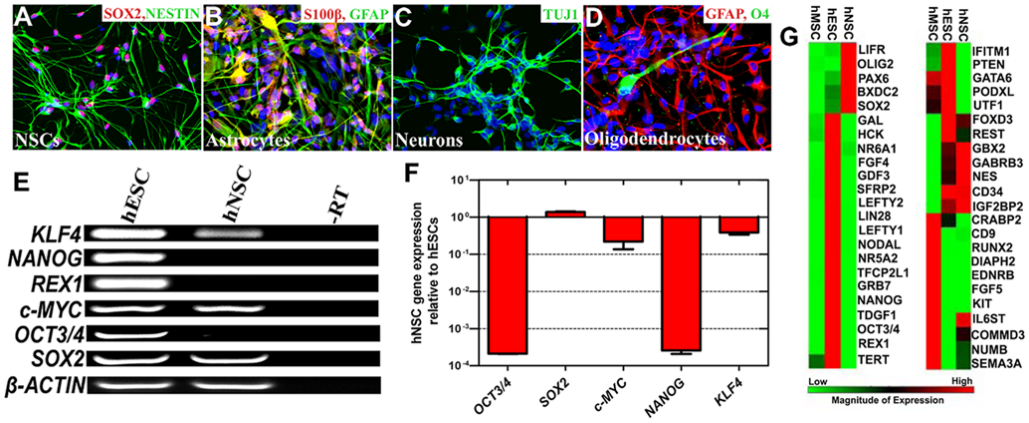
Human NSCs express several genes involved in the maintenance of pluripotency. Human NSCs express SOX2 and NESTIN (A) and are tri-potent, which can differentiate into astrocytes shown by GFAP (green) and S100-Beta (red) staining (B), neurons shown by TUJ1 staining (green) (C), and oligodendrocytes shown by O4 staining (green) (D). (E) Semi-quantitative RT-PCR analysis of key pluripotency genes expressed in human ES cells and several lacking in human NSCs. (F) Quantitative RT-PCR analysis of key pluripotency genes in human NSCs relative to hESCs. (G) Heat map derived from quantitative RT-PCR depicting relative expression of multiple genes associated with pluripotency comparing human MSCs, NSCs, and ESCs.

In contrast, hNSCs differ greatly from hESCs in their growth properties, differentiation potential, and gene expression profile. As shown in 1A, similar to hESCs, hNSCs express high levels of SOX2, which is considered a key regulator of pluripotency [19], and NESTIN, a protein specific to NSCs [21]–[23]. Human NSCs have been generally considered to have limited differentiation with the capacity to differentiate into only neurons, astrocytes, and oligodendrocytes under permissive conditions, as we have observed (Figure 1B–D). In addition, the self-renewal capacity of hNSCs is more limited than hESCs [17]. To further investigate additional similarities between hNSCs and hESCs at the transcriptional level, we analyzed the expression of key genes involved in hESC self-renewal and pluripotency utilizing semi-quantitative RT-PCR analysis. As shown in Figure 1E, all key genes involved in maintaining pluripotency were expressed in hESCs, as expected. In contrast, hNSCs were shown to express a more limited subset of factors, which included SOX2, c-MYC, and KLF4. To quantitatively determine differences between the expression levels of these genes in hNSCs and hESCs, we utilized real time RT-PCR. Our results showed that hNSCs express 1.4 fold higher levels of SOX2, fourfold lower levels of c-MYC, and threefold lower levels of KLF4 relative to hESC expression levels. In comparison, mouse NSCs were reported to express twofold higher levels of Sox2, an eightfold lower level of Klf4, and a tenfold higher level of c-Myc expression relative to mouse ESC expression levels [14].

We further extended our analysis of pluripotency gene expression to a much larger scale to potentially identify similar signaling cascades that might be conserved between hNSCs and hESCs. We utilized quantitative RT-PCR to measure 46 genes that are expressed in hESCs, which function as cytokines, growth factors, and other signaling molecules. In addition to analyzing pluripotency gene expression in hESCs and hNSCs, we also performed our analysis on human mesenchymal stem cells (hMSCs), which are also considered to have a limited differentiation potential. As depicted in a heat map format in Figure 1G, most of the genes analyzed are expressed at higher levels in hESCs compared to hNSCs or hMSCs, as expected. Interestingly, hNSCs highly expressed receptors involved in STAT signal transduction such as LIFR and IL6ST. Indeed, it has been demonstrated that LIF promotes self-renewal of human neural stem cells *in vitro*, though STAT signaling is insufficient to promote self-renewal in hESCs [24]–[26]. In sum, analysis of genes involved in pluripotency in hNSCs has revealed these cells are primed with several factors such as SOX2, c-MYC, and KLF4, which might support more readily their induction into a pluripotent state.

### iPS Cells Can Be Generated from Human NSCs by Exogenous Expression of OCT3/4 and KLF4

**Figure 2:**
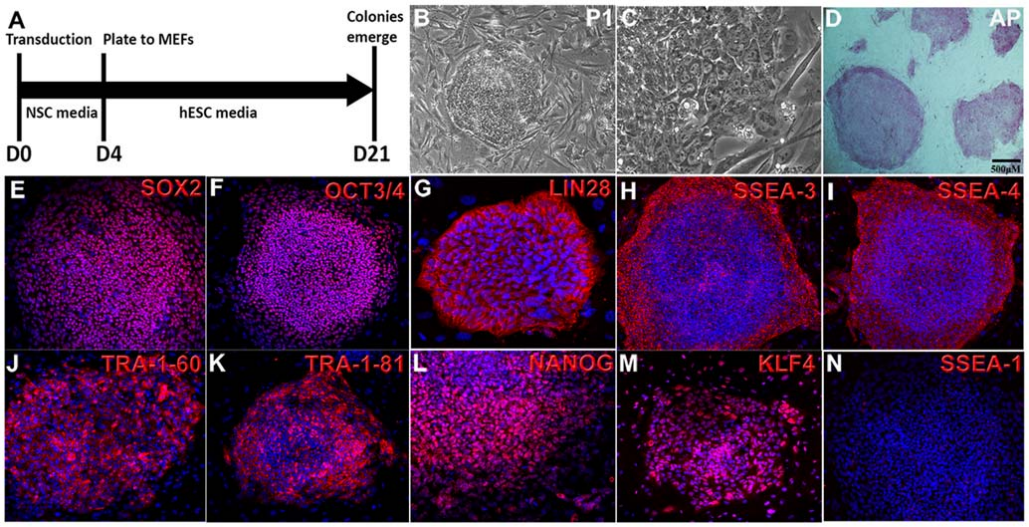
Human iPS cells can be generated from human NSCs. (A) Schematic depicting the strategy to induce pluripotency in human NSCs. (B) After 21 days iPSC colonies emerge, which are molecularly indistinguishable from hESC colonies under low and high magnification (C) and stain positive for alkaline phosphatase (D). (E–M) Extensive immunocytochemical analysis of genes expressed human NSC-derived iPS cells, which are also expressed in hESCs except for SSEA-1 (N), which is only expressed in mouse ES cells.

Since we observed robust expression of SOX2 and modest expression of c-MYC and KLF4 in our human NSC cultures, we tested if ectopic expression of OCT3/4 per se would be sufficient to convert them from multipotent to pluripotent stem cells. Furthermore, it was recently demonstrated that Oct3/4 expression in mouse NSCs was sufficient to convert them into iPS cells, suggesting this strategy might function in hNSCs. We infected 1×10^6^ hNSCs with OCT3/4 retrovirus, and after multiple attempts and long incubation periods greater than two months, we did not observe any colonies. This inability to reprogram human NSCs with OCT3/4 alone might indicate possible differences between mouse and human NSCs. To rule out whether this might be due to low targeting efficiencies, we performed immunocytochemistry against OCT3/4 and observed an 80% infection rate (data not shown), suggesting this was not an inability to deliver OCT3/4 to hNSCs. Since KLF4 has been shown to play a critical role in chromatin remodeling and maintaining self-renewal in hESCs [27], [28], and it was shown to support iPS cell induction in conjunction with Oct3/4 from mouse NSCs [14], [16], we introduced it into our iPS induction strategy. We achieved a targeting efficiency of approximately 80% with both retroviruses containing OCT3/4 and KLF4. The day after retroviral transduction, cells were grown in NSC media for four days following a change to hESC media to support iPS cell growth (Figure 2A). Within ten days we observed localized areas of dense cell growth and within three weeks, we observed cells that were morphologically similar to hESCs (Figure 2B, C). Based on the percentage of cells that were infected with both OCT3/4 and KLF4 at 80%, we calculated our reprogramming efficiency to be approximately 0.01% (80 colonies from 0.8×10^6^ cells infected with both OCT3/4 and KLF4), which was elevenfold less efficient than iPS induction from mouse NSCs with Oct3/4 and Klf4. This may be due to lower endogenous expression of c-MYC in human NSCs compared to mouse NSCs [14]. The efficiencies of reprogramming human dermal and neonatal foreskin fibroblasts to iPS cells utilizing OCT4, SOX2, KLF4, and c-MYC have been shown to range between 0.01–0.02% [6], [9], [10], [12] which is quite similar to our reprogramming efficiencies on human NSCs with OCT3/4 and KLF4. We picked seven iPS clones that were morphologically similar to hES cells, and of those three iPS lines were generated. To verify whether the retroviral KLF4 and OCT3/4 transgenes were present in our iPS clones, we performed PCR analysis and observed both transgenes were integrated in all clones we analyzed (Figure S1A). In addition, iPS cells stained positive for alkaline phosphatase (AP) activity, which is a prototypical marker for ES cells (Figure 2D). We also found expression of a wide panel of markers expressed in hESCs in our iPS cells, which include OCT3/4, SSEA-3, TRA-1-60, TRA-1-81, NANOG, KLF4, and LIN28 (Figure 2E–M). In contrast, SSEA-1 staining was not observed, as expected, since it is marker exclusive to mouse ES cells (Figure 2N). All iPS lines (iPS1-iPS3) showed the full complement of pluripotency markers utilized.

**Figure 3:**
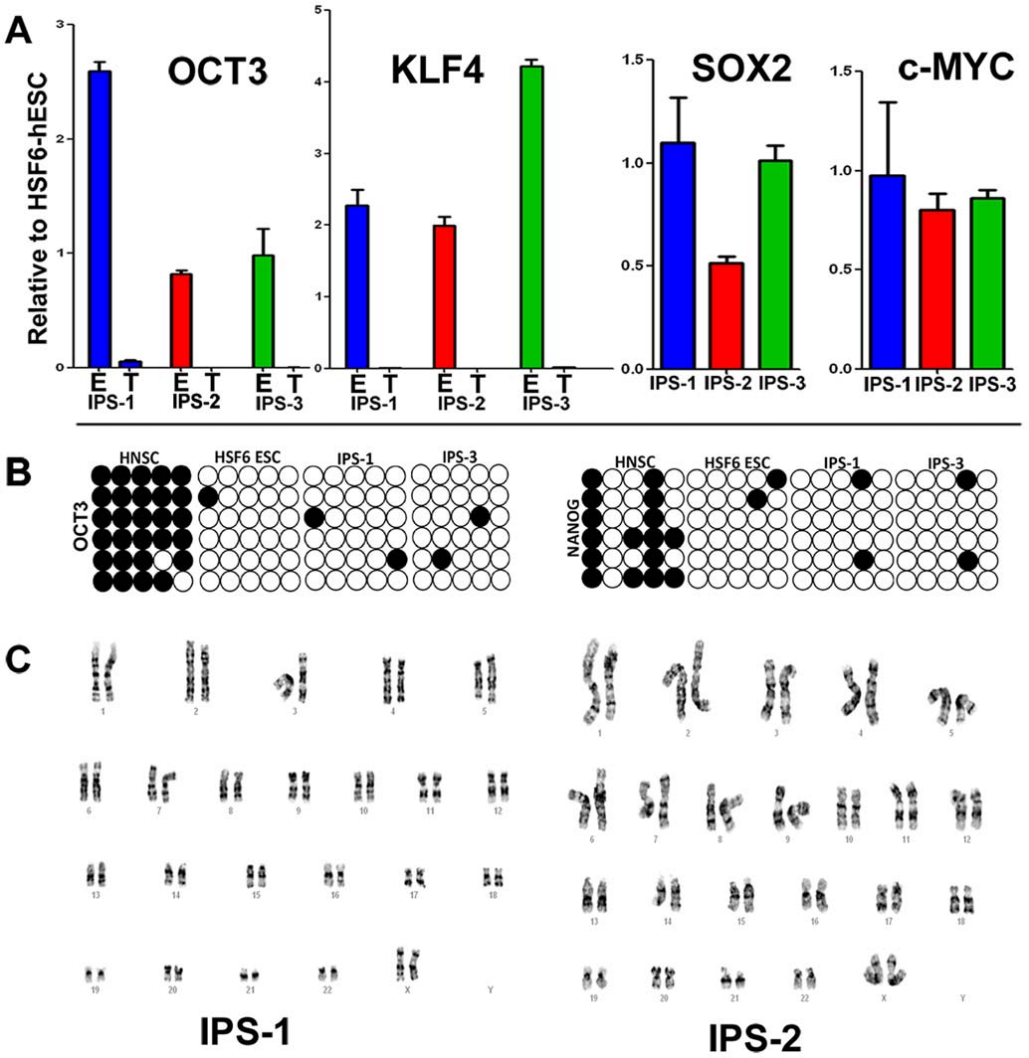
Human NSC-derived iPS cells are genetically similar to hESCs. (A) Quantitiative RT-PCR analysis of key pluripotency inducing genes from endogenous (shown as “E”) loci or transgenic (shown as “T”) loci normalized to hESC gene expression. (B) Bisulfite sequence analysis of the human OCT3 and NANOG promoters in hESCs and multiple iPS clones. Unfilled or filled circles represent unmethylated or methylated CpGs, respectively. (C) Karyotype analysis of multiple iPS clones.

To further confirm the authenticity of our iPS clones, we performed quantitative RT-PCR on four major pluripotency inducing genes, OCT3/4, KLF4, SOX2, and c-MYC, in three of our iPS clones. We also performed qRT-PCR to determine whether the retroviral OCT3/4 and KLF4 transgenes were silenced, which has been shown to be a reliable measure for efficient reprogramming of somatic cells into pluripotency [29]. As shown in Figure 3A, all iPS lines tested showed similar levels of these factors compared to hESC cells. In addition, all lines showed nearly complete silencing of the transgenes for both OCT3/4 and KLF4 (Figure 3A).

A more stringent test was utilized to determine whether reprogramming human NSCs was accompanied by demethylation of the OCT3/4 and NANOG promoters, both of which must be active to maintain pluripotency. To do this, we performed bisulfite sequencing to evaluate the methylation status of these promoters in several of our iPS lines. Consistent with our earlier findings analyzing OCT3/4 and NANOG gene expression, both promoters were hypermethylated and hypomethylated in hNSCs and hESCs, respectively (Figure 3B). All of these data highly support that iPS cells derived from hNSCs can be reprogrammed to a hESC-like state at the molecular level. This reprogramming to an ESC like state was also accompanied by an increase in proliferation as shown by flow cytometry analysis (FACS). Quantification of this data showed that hNSC-derived iPS cells were more similar to hESCs in the percentage of cells in the G2/M phase of the cell cycle than were hNSCs (Figure S1b). This increase in proliferation was not due to transformation in culture since karyotypic analysis of all iPS lines analyzed (iPS1-2) revealed a normal number of chromosomes (46XX) without gross abnormalities (Figure 3C).

### Human NSC-derived iPS Cells Can Differentiate into All Three Germ Lineages *In vitro* and *In vivo*

**Figure 4:**
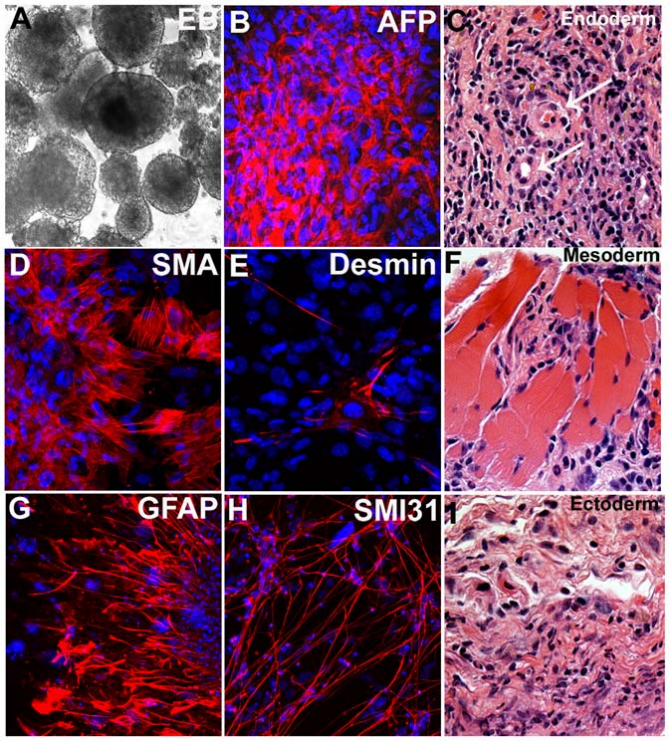
Human NSC-derived iPS cells can form all three germ lineages both *in vitro* and *in vivo*. (A) iPS cells can form embryoid bodies (EB) and can differentiate *in vitro* to endoderm shown by alpha fetoprotein (AFP) (B), mesoderm shown by smooth muscle actin (SMA) and desmin (D,E), and ectoderm shown by GFAP and SMI31 staining (G,H). iPS cells injected into athymic nude mice form teratomas and differentiate into all germ lineages *in vivo* (C,F,I).

Pluripotent stem cells are capable of differentiating into all three germ lineages of the human body, and we thus investigated the developmental potential of our i-PS cells to determine whether they could differentiate into ectoderm, endoderm, and mesoderm. We differentiated our iPS cells as embryoid bodies (EB) (Figure 4A) in the presence of FBS to induce endodermal and mesodermal fates, and in the presence of retinoic acid to induce an ectodermal fate, and after ten days allowed them to adhere to tissue culture dishes. After the EBs adhered, we analyzed our cultures for different germ layer markers by immunocytochemical analysis. As shown in Figure 4, all three germ layers developed in our cultures as shown by alphafetoprotein (AFP) for endoderm (Figure 4B), smooth muscle actin (SMA) and desmin for mesoderm (Figure 4D, E), glial fibrillary acid protein (GFAP) and SMI31 for ectoderm (Figure 4G, H). To determine the *in vivo* developmental potential of our iPS lines, we injected iPS-1 and iPS-3 lines into athymic nude mice to induce teratomas. The teratomas analyzed were shown to contain muscle tissues, which are derivatives of mesoderm, primitive duct formations (indicated by arrows), which are derivatives of endoderm, and epidermal structures, which are derivatives of ectoderm (Figure 4C, F, I). All iPS lines tested (iPS1, 3) gave rise to teratomas with all three germ layers. These data support the developmental potential of the iPS cells showing that they have the ability to give rise to all three cell lineages both *in vitro* and *in vivo*, which are critical attributes of pluripotent stem cells.

## Discussion

Here we have identified another source of human cells that can be efficiently reprogrammed into human iPS cells by the ectopic exposure of only two factors, namely OCT3/4 and KLF4. The mechanism of this two-factor reprogramming is probably largely due to the intrinsic expression of SOX2 within human NSCs, which has been shown to a major protein involved in maintaining pluripotency in hESCs. It is interesting that OCT3/4 was not sufficient to convert human NSCs into iPS cells utilizing our techniques. This could reflect intrinsic differences between mouse and human NSCs, but might still be attainable by altering some experimental parameters such as infecting more target cells, extending culturing conditions, and incubating target cells with a rho-kinase inhibitor small molecule, shown to enhance survival of human ES cells [30], [31]. Through extensive examination, we have shown that human NSC-derived iPS cells are phenotypically identical to hESCs by all pluripotential markers analyzed and for their ability to differentiate into all three major germ lineages.

The utilization of human NSCs as a potential source for reprogramming into iPS cells offers multiple advantages. Since the reprogramming process requires only two factors, the chance of insertional mutagenesis is minimized. Furthermore, obviating the requirement for c-MYC reduces the risk of inducing cancerous cells. In addition, since two factors are sufficient for human iPS induction from NSCs, it may be possible to substitute one or both of these factors with small molecules that activate OCT3/4 or KLF4 gene expression. Indeed, a recent report has demonstrated that the small molecule, kenpaullone could replace KLF4 in reprogramming mouse fibroblasts into iPS cells [32]. This report suggests it may be possible to reprogram human NSCs into iPS cells by OCT3/4 and kenpaullone, further minimizing the undesired effects of utilizing retroviral vectors. Another advantage of utilizing human NSCs is that they endogenously express SOX2, KLF4, and c-MYC, which provides a basis to analyze the mechanism of iPS induction with two factors.

Research on induced pluripotency has been rapidly expanding providing more efficient means to generate iPS cells. Indeed, a plethora of research reports has implicated the master guardian of the genome, p53, as being a major hindrance to the iPS induction process [33]–[38]. By impairing the p53 pathway on multiple levels, researchers were able to dramatically enhance the iPS cell induction up to 100 fold. By impairing the p53 pathway in hNSCs, it is probable that this will also increase the rate of IPS cell induction from these cells. In sum, human NSCs represent an invaluable source of cells to investigate human iPS induction, and also represent a potential source of cells for deriving patient-specific pluripotent stem cells for modeling human disease.

## Supporting Information

Figure S1 Multiple iPS lines show integration of both the OCT3/4 and KLF4 transgenes and progress through the cell cycle similarly to hESCs. (A) Genotype analysis of multiple iPS lines for OCT3/4 and KLF4 transgenes. (B) FACs analysis of the cell cycle in proliferating hNSCs, hESCs, and iPS cells with a chart showing percentages of cell types in the G2/M phase of the cell cycle. Supplemental Table 1. Information regarding the primary antibodies utilized in immunofluorescence analysis and primers utilized in RT-PCR, genotyping, and bisulfite sequencing analyses.
(0.95 MB TIF)

Table S1 Table S1. Information regarding the primary antibodies utilized in immunofluorescence analysis and primers utilized in RT-PCR, genotyping, and bisulfite sequencing analyses.
(0.05 MB DOC)
