# Supplementary material for: Two Factor Reprogramming of Human Neural Stem Cells into Pluripotency
Source: PLoS One. 2009 Sep 18;4(9):e7044. doi: 10.1371/journal.pone.0007044 (PMC2739296; doi:10.1371/journal.pone.0007044)
Supplement: Table S1 — Information regarding the primary antibodies utilized in immunofluorescence analysis and primers utilized in RT-PCR, genotyping, and bisulfite sequencing analyses. (0.05 MB DOC) [file pone.0007044.s002.doc]

| **Antibody** | **Dilution** | **Species** | **Company** |
| --- | --- | --- | --- |
|  |  |  |  |
| SMI31 | 1:200 | Rabbit | Millipore |
| AFP | 1:200 | Mouse | DSHB |
| GFAP | 1:200 | Rabbit | Santa Cruz |
| TRA-1-81 | 1:200 | Rabbit | Abcam |
| TRA-1-60 | 1:200 | Mouse | DSHB |
| SSEA-3 | 1:200 | Mouse | DSHB |
| SSEA-4 | 1:100 | Mouse | DSHB |
| SSEA-1 | 1:1000 | Mouse | Covance |
| NESTIN | 1:200 | Goat | Santa Cruz |
| OCT3/4 | 1:200 | Goat | Millipore |
| NANOG | 1:200 | Rabbit | Millipore |
| SOX2 | 1:500 | Rabbit | Millipore |
| DESMIN | 1:500 | Mouse | DSHB |
| SMA | 1:200 | Rabbit | Millipore |
| α-ACTININ | 1:1000 | Mouse | Sigma |
| S100-BETA | 1:200 | N/A | Invitrogen |
| TUJ1 | 1:1000 | Rabbit | Covance |
| O4 | 1:100 | Mouse | Thermo Scientific |

**Supplemental Table 1**

**Immunofluorescence**

**RT-PCR and Bisulfite analysis**

| **Gene** | **Forward Primer** | **Reverse Primer** |
| --- | --- | --- |
|  |  |  |
| REX1 | gtggatgcgcacgtgcgtacgc | ctggaggaatacctggcattg |
| OCT3/4 | gagcaaaacccggaggagt | ttctctttcgggcctgcac |
| NANOG | gcttgccttgctttgaagca | ttcttgaccgggaccttgtc |
| SOX2 | cggccccggcggaaaaccaa | tcggcgccggggagatacat |
| β-ACTIN | GGAGAAGATTTGGCACCACA | GAGTCCATCACAATGCCAGT |
| KLF4 | GGAACCGTGGCTCGGCCTCATTTC | AAAAGGCCTCACACATCTG |
| C-MYC | tttcaggggcgggtgcattgtagt | aggggcggtgcgatcagaggaagg |
| NANOG-F  Bisulfite | GCCCCAGGGTTATGAGACTATCAC | CCGACAGAGCCCAGATGTAGTTCTT |
| NANOG-R  Bisulfite | TGCAGGTTTGGTTCCAGAACCGCC | GCCAGGCCTCCATGCTCCAGGGAG |
| OCT4-F  Bisulfite | cgtgcccgctccaaggtgtatca | cattgggctgctgctgctggagtt |
| OCT4-R  Bisulfite | gcacccggcgctctcctactcgt | tcgtcctcctcggggtcactgtcc |
